# Supplementary material for: Focal adhesion is associated with lithium response in bipolar disorder: evidence from a network-based multi-omics analysis
Source: Mol Psychiatry. 2023 Mar 29;29(1):6–19. doi: 10.1038/s41380-022-01909-9 (PMC11078741; doi:10.1038/s41380-022-01909-9)
Supplement: Supplementary file 4 — Supplementary Table 2 [file 41380_2022_1909_MOESM4_ESM.pdf]

Supplementary Table 2. Characteristics of patients used for the iPSC studies.

Original Study	Cell ID	Obtained Tissues	Ethnicity	Gender	Age at Sampling (yrs)	Age of Onset (yrs)	Main Diagnosis	Time to Treatment Failure/Relapse – *Prospective Study* (mos)	Termination Reasons – *Prospective Study**	Alda Score (total)	Episodes, Depression+ Mania (n)	Family History (Yes/No)	Lifetime Psychotic Features (Yes/No)
**Controls (*n*=6)**													
PGBD/VA	149-59	Fibroblast	Caucasian	Male	56	-	None	-	-	-	-	-	-
PGBD/VA	149-61	Fibroblast	Caucasian	Male	38	-	None	-	-	-	-	-	-
HALIFAX	J008	Lymphoblast	Caucasian	Male	25	-	-	-	-	-	-	-	-
HALIFAX	J009	Lymphoblast	Caucasian	Male	51	-	-	-	-	-	-	-	-
HALIFAX	J011	Lymphoblast	Caucasian	Male	62	-	-	-	-	-	-	-	-
HALIFAX	J012	Lymphoblast	Caucasian	Male	53	-	-	-	-	-	-	-	-
**BD Responders (*n*=6)**													
PGBD/VA	144-74	Fibroblast	Caucasian	Male	65	36	BD I	24	Completed	-	9	N	N
PGBD/VA	118-22	Fibroblast	Caucasian	Male	57	9	BD I	22	Relapse	-	30	N	N
PGBD/VA	116-92	Fibroblast	Caucasian	Male	59	6	BD I	23	Relapse	-	24	Y	N
HALIFAX	J005	Lymphoblast	Caucasian	Male	41	34	BD I	-	-	10/10	6	N	Y
HALIFAX	J007	Lymphoblast	Caucasian	Male	34	15	BD I	-	-	9/10	4	Y	N
HALIFAX	J010	Lymphoblast	Caucasian	Male	50	31	BD I	-	-	9/10	7	Y	Y
**BD Non-Responders (*n*=5)**													
PGBD/VA	96-91	Fibroblast	Caucasian	Male	54	15	BD I	3	Treatment failure	-	41	Y	Y
PGBD/VA	149-34	Fibroblast	Caucasian	Male	69	15	BD I	4	Treatment failure	-	11	Y	N
HALIFAX	J001	Lymphoblast	Caucasian	Male	51	35	BD I	-	-	3/10	3	N	Y
HALIFAX	J002	Lymphoblast	Caucasian	Male	58	22	BD I	-	-	1/10	10	N	N
HALIFAX	J004	Lymphoblast	Caucasian	Male	40	24	BD I	-	-	0/10	31	Y	N

See additional details of the PGBD/VA and HALIFAX studies in **Supplemental Methods**.
***Completed** refers to those subjects who completed the 24-month maintenance follow-up phase without relapse; **Relapse** refers to those who completed the initial 4-month stabilization phase but relapsed in the maintenance phase follow-up; **Treatment failure** refers to those who failed to achieve remission during the initial 4-month stabilization phase.
HALIFAX, a study from Dalhouise University; PGBD/VA, a study from the Pharmacogenomics of Bipolar Disorder study and the Veterans Affairs San Diego Healthcare System.
